# Unseen Burdens: How ACEs and Citizenship Status Shape Health in Aging Latinx Populations

**DOI:** 10.1093/geroni/igaf122.1657

**Published:** 2025-12-31

**Authors:** Alein Haro-Ramos

**Affiliations:** University of California, Irvine, Irvine, California, United States

## Abstract

**Background and Objectives:**

Citizenship (or the lack thereof) and adverse childhood experiences (ACEs) are both independent predictors of poor self-rated health (SRH), but we know little about their cumulative effects among older Latinx adults. Using a life course perspective, we examine 1) the relationship between ACEs, citizenship status, and poor SRH and 2) whether race-ethnicity-citizenship status combinations modify the association between ACEs and SRH.

**Research Design and Methods:**

Latinx and White adult respondents age 50+ were selected from the 2021 and 2022 California Health Interview Survey (n = 20,491). Generalized linear models (logit link, binomial family) estimated the relationship between ethnicity-citizenship status, ACEs, and SRH. We use an interaction between ethnicity-citizenship and ACEs to test for a moderating effect on SRH.

**Results:**

High ACE exposure and noncitizen status were each significant predictors of poor SRH, adjusting for covariates. In the mutually adjusted model, including ACEs and citizenship status, the magnitude of the coefficient for naturalized and noncitizen Latinx adults and for each ACE score increased. The association between ACEs and poor SRH was strongest among noncitizen Latinx adults.

**Discussion and Implications:**

ACEs and citizenship status are significant predictors of poor SRH, and the relationship between ACEs and SRH is pronounced among noncitizen Latinx older adults. Our findings underscore the importance of addressing early and later life experiences to understand and improve the health and well-being of older Latinx individuals, particularly those lacking citizenship.

